# Safety and Efficacy of Dolutegravir Plus Rilpivirine in Treatment-Experienced HIV-Infected Patients: The DORIVIR Study

**DOI:** 10.1177/2325958218760847

**Published:** 2018-03-12

**Authors:** Rosario Palacios, M. Mayorga, C. M. González-Domenech, C. Hidalgo-Tenorio, C. Gálvez, L. Muñoz-Medina, J. de la Torre, A. Lozano, M. Castaño, M. Omar, Jesús Santos

**Affiliations:** 1 UGC Enfermedades Infecciosas y Microbiología, Hospital Virgen de la Victoria, Málaga, Spain; 2 UGC Enfermedades Infecciosas y Microbiología, Hospital Carlos Haya, Málaga, Spain; 3 Servicio de Enfermedades Infecciosas, Complejo Hospitalario Universitario Granada, Granada, Spain; 4 UGC Enfermedades Infecciosas, Hospital Torrecárdenas, Málaga, Spain; 5 UGC Enfermedades Infecciosas, Hospital Costa del Sol, Málaga, Spain; 6 UGC Enfermedades Infecciosas, Hospital de Poniente, Almería, Spain; 7 UGC Enfermedades Infecciosas, Complejo hospitalario de Jaén, Jaén, Spain

**Keywords:** safety, efficacy, dolutegravir/rilpivirine, ART switching, lipid profile improvement

## Abstract

**Objectives:**

To analyze the efficacy and safety of dolutegravir/rilpivirine (DTG/RPV) in HIV-infected patients who switched from any other antiretroviral therapy (ART).

**Methods:**

Open-label, multicenter study including patients who switched to DTG/RPV between February 2015 and February 2016. Efficacy (HIV RNA <50 copies/mL), adverse events, and metabolic changes at 24 weeks were analyzed.

**Results:**

A total of 104 participants were included, who switched for the following reasons: toxicity/intolerance (42.3%), convenience (27.8%), and drug interactions (17.3%). Prior regimens are protease inhibitor (56.7%), integrase strand transfer inhibitor (26.9%), and non-nucleoside reverse transcriptase inhibitor (16.3%). Efficacy at 24 weeks was 88.4% (intention to treat) and 96.8% (per protocol). Triglyceride levels were reduced, on average, by 12.7% and a mean decrease of 9.0% in the glomerular filtration rate was observed as well (*P* values of .003 and .002, respectively), whereas total cholesterol, HDL cholesterol, LDL cholesterol, creatinine, and glutamic-pyruvic transaminase remained unchanged. No patient discontinued due to adverse events.

**Conclusions:**

Dolutegravir/RPV is effective and safe in long-term HIV-infected patients under any prior ART. Toxicity, convenience, and interactions were the main reasons for changing. At 24 weeks, the lipid profile improved with a decrease in triglycerides.

## Introduction

The use of dolutegravir (DTG) and rilpivirine (RPV) as a 2-drug nucleoside reverse transcriptase inhibitor (NRTI)- and protease inhibitor (PI)-sparing regimen is very well tolerated, with rare adverse events or drug interactions and with no metabolic, liver, kidney, or bone toxicity. It is therefore a good option as a simplification therapy,^1-5^ especially with the coming DTG/RPV formulation in a single tablet (ClinicalTrials.gov Identifier: NCT02373930). Two ongoing phase III randomized clinical trials (ClinicalTrials.gov Identifier: NCT02429791 and ClinicalTrials.gov Identifier: NCT02422797, SWORD-1 and SWORD-2) are aimed at assessing the efficacy and safety of switching to DTG/RPV from a prior antiretroviral therapy (ART) based on 2 NRTIs plus a PI, an integrase strand transfer inhibitor or non-NRTI. These clinical trials have shown noninferior antiviral activity for the mentioned switch to a once-daily regimen compared to the continuation of a triple therapy regimen up to week 48.^6^ Nevertheless, data related to treatment with DTG/RPV in usual clinical practice are few and derived from just 3 studies: 11 patients among 31 cases under different dual therapies based on DTG,^7^ 132 patients from 8 Italian centers,^8^ and 106 cases, all on simplification treatment (French Dat’AIDS cohort).^9^ None of these studies assessed the impact of the switching strategy on the lipid profile though. Our study aimed to analyze the efficacy and safety of the dual combination DTG/RPV in HIV-infected patients switching from any previous ART to this regimen.

## Patients and Methods

From a cohort of 7270 HIV-infected patients ≥18 years of age being regularly followed at 7 hospitals in Andalusia (southern Spain), we retrospectively selected all those who switched to DTG/RPV from any ART between February 2015 and February 2016. This was therefore an open-label, noncontrolled, multicenter study. We collected epidemiological, clinical, and therapeutic data, as well as the reason for switching. The primary end point was the proportion of patients with HIV RNA <50 copies/mL at 24 weeks after switching (missing = failure). If the patient presented virologic failure, genotype resistance tests were performed. Immunologic aspects were also considered at this time (after 24 weeks). Secondary end points were adverse events due to this dual therapy and the associated rate of discontinuation at 24 weeks after switching. Adverse events and deaths were reported to the ethics committees of the centers involved and the relevant authorities.

We also assessed metabolic changes resulting from switching, taking measurements at baseline and after 24 weeks of plasma lipids (triglycerides, total cholesterol, high-density lipoprotein cholesterol, and low-density lipoprotein cholesterol), glutamic–pyruvic transaminase (GPT), and renal parameters (both creatinine value and estimated glomerular filtration rate [eGFR] to a better assessment of renal function).

This study was approved by the ethics committee of the coordinating center and supported by the Andalusian Society of Infectious Diseases (SAEI), which also approved it, registering it with the code SAEI 00/0067.

Data from all the patients enrolled were analyzed statistically with SPSS, version 17.0 (SPSS). A descriptive analysis of the variables was estimated with a 95% confidence interval. The continuous variables were calculated as the mean (with standard deviation) or median (interquartile range) depending on their distribution within the cohort, whereas the qualitative variables were expressed as frequencies or percentages. Student *t* test was used in paired samples to contrast continuous variables at baseline and 24 weeks after switching.

## Results

Although 105 patients started DTG/RPV during the period considered, one was naive and was therefore discarded, leaving 104 who fulfilled the inclusion criteria (≥18 years of age and switching to the abovementioned regimen within the study period): 82 (78.8%) with virologic suppression and 22 (21.1%) without virologic suppression (8 virologic failure and 14 restarting ART). The median age of the cohort was 51.0 years (range: 47.6-56.0), and 70.2% were male patients. The prevalence of hepatitis C virus antibodies was 28.8%, but only a positive case (0.9%) of hepatitis B surface antigen was found. The most frequent reasons for the switch to DTG/RPV were toxicity/intolerance (42.3%), convenience (27.8%), and drug interactions (17.3%). Table 1 summarizes these and other baseline clinical and epidemiological characteristics of the study patients. At 24 weeks after switching to DTG/RPV, 95 (91.3%) patients remained under the same therapeutic regimen, with an increase in the CD4 T-cell count (552 versus 622 cells/mm^3^; *P* = .008). Eight patients were lost to follow-up and another patient discontinued the dual combination because of lack of viral suppression at week 24; this latter case was previously being treated with emtricitabine/tenofovir plus raltegravir and restarted ART with DTG/RPV due to toxicity of the mentioned regimen. At week 24, 92 participants showed HIV RNA <50 copies/mL, giving an efficacy of 88.4% by intention-to-treat analysis and 96.8% by per-protocol analysis. Three cases had a detectable viral RNA load (values of 532, 316, and 75 copies/mL, respectively), though they were not considered virologic failures. Two of these unsuppressed patients started DTG/RPV as a salvage therapy (9.0% of 22% cases). Finally, a mean decrease of 12.7% (*P* = .003) and 9.0% (*P* = .002) in the levels for triglycerides and eGFR was observed at week 24, respectively, with no changes in total cholesterol or its fractions, GPT, or creatinine (Table 2). None of the enrolled patients discontinued DTG/RPV due to adverse events. Regarding triglycerides levels, this reduction was only statistically significant (184 versus 134, *P* < .0001; mean decrease −49.7 mg/dL) in the subset of patients with a PI in their prior regimen, but not in the rest of the cohort (195 versus 173, *P* = .3; mean decrease −22.2 mg/dL).

**Table 1.** Characteristics of the Patients Included.^a^

Variables	
No. of patients	104
Male gender	73 (70.2)
Age (years)	51.0 (47.6-56.0)
HIV transmission category	
MSM	34 (32.7)
Heterosexual	31 (29.8)
IDU	37 (35.6)
Other	2 (1.9)
Months since HIV diagnosis	238.5 (140.4-288.9)
AIDS cases	45 (43.3)
Nadir CD4 count T cells/mm^3^	140 (61-242)
CD4 count T cells/mm^3^ at switching time	536 (316-735)
Months on previous ART	26.4 (7.8-68.2)
Previous ART	
NNRTI	17 (16.3)
PI	59 (56.7)
INSTI	28 (26.9)
Reasons for switching to DTG/RPV	
Toxicity/intolerance	44 (42.3)
Convenience	29 (27.8)
Drug interactions	18 (17.3)
Other	13 (12.5)

Abbreviations: ART, antiretroviral therapy; DTG, dolutegravir; IDU, intravenous drug user; INSTI, integrase strand transfer inhibitor; MSM, men who have sex with men; NNRTI, non-nucleoside reverse transcriptase inhibitor; PI, protease inhibitor; RPV, rilpivirine.

^a^The continuous variables are expressed as the median (interquartile range, IQR) and the qualitative variables in n (%).

**Table 2.** Comparison of Analytical Parameters between Baseline and 24 Weeks after Switching to DTG/RPV Combination.^a^

Variables	Baseline	24 Weeks after	*P* Value
Lipid profile			
Cholesterol (mg/dL)	183 (153-223)	177 (152-214)	.2
HDL-C (mg/dL)	46 (36-57)	46 (38-54)	.7
LDL-C (mg/dL)	105 (82-138)	104 (82-132)	.3
Triglycerides (mg/dL)	149 (107-224)	130 (86-175)	.003
TC: HDL-C	4.09 (3.3-5.2)	3.9 (3.2-4.7)	.3
Renal profile			
Creatinine (mg/dL)	0.9 (0.7-1.1)	1.0 (0.8-1.2)	.2
eGFR (mg/mL/min)^b^	88.0 (70.5-90.0)	80.0 (64.9-90.0)	.002
GPT (IU/L)	28 (20-40)	29 (20-39)	.6

Abbreviations: DTG, dolutegravir; eGFR, estimated glomerular filtration rate; GPT, glutamic–pyruvic transaminase; HDL-C, high-density lipoprotein cholesterol; LDL-C, low-density lipoprotein cholesterol; RPV, rilpivirine; TC, total cholesterol.

^a^The continuous variables are expressed as the mean (interquartile range, IQR) and the qualitative variables in n (%).

^b^Estimated according to the Chronic Kidney Disease–Epidemiology Collaboration (CKD-EPI) equation.

## Discussion

This study provides results confirming the efficacy and safety of the dual combination DTG/RPV in daily clinical practice for long-term HIV-infected patients, who had also had extensive experience with ART. Most of their previous regimens were more complex. The main reasons for switching were toxicity/intolerance, convenience, and drug interactions, and most of these patients were virologically suppressed at the time of switching. Nevertheless, a significant percentage of cases (21.1%) presented a detectable viral load when starting DTG/RPV either because of virologic failure or discontinuation of their former ART. Among these patients starting DTG/RPV as a rescue treatment, only 2 (9.0%) patients showed a detectable viral load at week 24, but they were not labeled as failures since they eventually became virologically suppressed during further follow-up. These patients had few therapeutic options, so the dual combination DTG/RPV, with a proven efficacy in them as well, differentiates this regimen from other NTRI-sparing therapies mainly tested in already virologically suppressed patients. Additionally, there were another patient not suppressed after 24 weeks but later in his follow-up, so we can assert that none of the patients from our cohort discontinued because of virologic failure, as observed in other studies.^7,8^ Only 1 patient was withdrawn from the study due to a detectable viral load at week 4, but this period of time is not enough to assess efficacy though.

The regimen DTG/RPV was very well tolerated, without any patient from our study discontinuing because of adverse events, unlike the low tolerability previously described for DTG.^9-11^ In addition, as far as we know, this is the first study reporting an improvement in the lipid profile after switching to the dual combination. This is due to over half the patients in our cohort were under a prior regimen containing a PI. In fact, the comparison of the triglycerides levels at baseline and after 24 weeks for patients with and without a PI in the previous ART only found a statistically significant decrease in the first group. Finally, we should note the lack of a control group and the heterogeneity of our cohort as the main limitations of our study. In conclusion, the dual combination DTG/RPV was effective, safe, and well tolerated in long-term treatment-experienced HIV-infected patients in daily clinical practice.
